# Joint Deep Model with Multi-Level Attention and Hybrid-Prediction for Recommendation †

**DOI:** 10.3390/e21020143

**Published:** 2019-02-03

**Authors:** Zhipeng Lin, Yuhua Tang, Yongjun Zhang

**Affiliations:** 1 State Key Laboratory of High Performance Computing, College of Computer, National University of Defense Technology, Changsha 410073, China; 2 National Innovation Institute of Defense Technology, Beijing 100089, China

**Keywords:** Recommender System, deep learning, attention-based mechanism, Factorization Machine

## Abstract

The Recommender System (RS) has obtained a pivotal role in e-commerce. To improve the performance of RS, review text information has been extensively utilized. However, it is still a challenge for RS to extract the most informative feature from a tremendous amount of reviews. Another significant issue is the modeling of user–item interaction, which is rarely considered to capture high- and low-order interactions simultaneously. In this paper, we design a multi-level attention mechanism to learn the usefulness of reviews and the significance of words by Deep Neural Networks (DNN). In addition, we develop a hybrid prediction structure that integrates Factorization Machine (FM) and DNN to model low-order user–item interactions as in FM and capture the high-order interactions as in DNN. Based on these two designs, we build a Multi-level Attentional and Hybrid-prediction-based Recommender (MAHR) model for recommendation. Extensive experiments on Amazon and Yelp datasets showed that our approach provides more accurate recommendations than the state-of-the-art recommendation approaches. Furthermore, the verification experiments and explainability study, including the visualization of attention modules and the review-usefulness prediction test, also validated the reasonability of our multi-level attention mechanism and hybrid prediction.

## 1. Introduction

With dramatically increasing information available online, the Recommender System (RS) is playing an increasingly important role in many customer-oriented applications and services to alleviate information overload. Facing information explosion, Internet users rely on RS to filter out uninteresting information and direct access helpful information in classical application scenarios such as listening music, watching movie and online shopping. Meanwhile, recommender systems can also bring traffic and make profit for online companies. For example, it is reported in [1] that recommender systems in Netflix accounted for about 80% of movies watched and generated immense economic value of more than $1 billion (this paper is an extended version to our previous conference publication [2]).

The key of recommender systems lays in the modeling of users’ preference [3]. Collaborative Filtering (CF) plays a pivotal role in modern recommender systems and many promising approaches in the literature adopt the idea of CF to predict the users’ preference [4]. The basic assumption of CF is that the users who have similar behavior such as historical clicking or rating tend to share similar preference and make the same choice. As a representative approach in CF, Probabilistic Matrix Factorization (PMF) has become the most famous and popular technique in RS [5]. PMF projects the uses and items into a common latent space and constructs the latent vector through factorization of rating matrix. The elements of a latent vector can be seen as contributions of underlying factors to the preference of a user or the property of an item. In PMF, the preference of users on items is modeled as the inner product of corresponding vectors.

Although the CF is a clever and compact idea, there are still several significant challenges in practical scenarios [6]. The first one comes from the sparsity of rating matrix [7]. The sparsity problem arises from the imbalance between the total number of items and the average number of items that a user can rate. Since there is plenty of missing value on the rating matrix, it is perplexing for CF to make prediction and give recommendation. In addition, CF techniques show weak performance in the ability to provide explanation. Some research [8,9] has pointed out that it is beneficial for RS to give some explanation for the sake of persuasion and convenience. Credible explanations on the recommended items can increase the users’ confidence and help them make decision more efficiently. Another dilemma is caused by the model of the interaction between users’ and items’ latent vectors. The traditional factorization approaches in RS, including Matrix Factorization (MF) [10], Factorization Machine (FM) [11] and Pairwise Interaction Tensor Factorization (PITF) [12], apply an inner product to model the pair-wise interaction between latent vectors and make rating prediction. However, as shown in [13,14], the simple inner product may miss high-order and non-linear parts in user–item interactions and lead to a deviation from original rating.

To address the sparsity problem and improve the explainability of RS, we explore the use of review text in RS. On most online commerce websites, users are encouraged to not only give a numerical rating on items but also write text reviews. The reviews contain rich information that can reflect the property and feature of items to some extent, which can give some suggestions and has certain reference significance to other users [15]. Recently, some studies have reported that importing the review text into RS can mitigate the effect of sparsity problem and strengthen the ability of providing explanation [16,17].

There are many models to extract the feature vector of review text in RS. However, it is still a challenge and significant issue for RS to extract the most informative feature from a tremendous amount of reviews. Existing RS models utilize reviews to boost the model of latent factor [17,18,19,20] and give explanation [21,22,23]. Although these methods have achieved impressive improvement, they still have some limitations in the design of attention mechanism. First, they do not model the contribution of each review and that of words in a single review text jointly. Some methods focus on the extraction of keywords and phrases but miss the contribution of each review [18,19], while some approaches only consider the usefulness of reviews [21]. Second, these methods mainly generate explanations through simple extractions of keywords or reviews, which may mislead users.

In real life, people are not only attracted to the keywords or phrases when reading review text but also focus on the useful reviews, especially those reflecting the property of items in plenty of reviews. Inspired by this common phenomenon, we design a multi-level attention mechanism for RS to extract most helpful information from all related reviews. The weights of words and reviews are defined as the informativeness and the usefulness of themselves. The weights of reviews is a extension of the definition of *usefulness* in [21] and the weights of words is an extension of the *weights* in [23]. To provide helpful information about items and give suggestions based on the interest of users, the weights are learned according to the preference of users or the property of items in two deep neural networks.

To capture the high-order and non-linear user–item interaction and avoid a deviation from original rating, we combine Deep Neural Network with FM [11]. As a popular method to extract feature representation, deep neural networks show the potential to handle the sophisticated feature interaction in the prediction of click-through rate (CTR) [14,24,25,26]. FM captures pair-wise interactions between latent factor vectors and shows impressive results. We extend the coupling of FM and deep neural networks in CTR [14,26] to the prediction layer of our RS model to capture both high-order and low-order interactions.

In this paper, we propose a novel Multi-level Attentional and Hybrid-prediction-based Recommender (MAHR) model. Specifically, we apply both word level and full-text level attention mechanisms on reviews through two deep neural networks, which extract feature vectors for each user and item. The word level attention mechanism is realized by a CNN text processor with a word-level attention layer, which learns the weights of words from a slip window. The full-text level attention mechanism applies a two-layered neural network with softmax function to measure the weights of reviews. In the prediction layer, we integrate FM and deep neural networks to model low-order user–item interactions as in FM and capture the high-order interactions as in deep neural networks. Experimental results show that our MAHR model consistently outperforms state-of-the-art methods. In addition, the results also show that and MAHR alleviates the sparsity problem and improve the explainability of RS.

The main contributions of this paper are:

- We propose a Multi-level Attentional and Hybrid-prediction-based Recommender (MAHR) model. To the best of our knowledge, we are the first to introduce a multi-level attention mechanism in RS, which alleviates the sparsity problem and improve the explainability of RS.
- In prediction layer, we hybrid FM and DNN to overcome the shortcomings of traditional handling of user–item interactions by simple inner product and capture both high-order and low-order interactions to achieve better prediction performance.
- Experimental results on benchmark datasets show that our MAHR model obtains better prediction results than the state-of-the-art models and the average relative improvement over PMF is up to 30.06%.

The extension of our journal paper to our previous conference publication [2] is as follows. In this article, we add the ID embedding of users and items to model the quality of users and items, which helps to identify users and items. In addition, the hybrid prediction layer is tuned to improve the memory efficiency. For the completeness of the model, we give a more precise description and analysis of our proposed method (Section 3). To make our experiments more solid, we extended our experiments from the comparison methods (CTR and D-Attn in Section 4.1) to the parameter sensitivity analysis (Figures in Section 4.3). We also detail the experimental methodology in Section 4.2. The discussion on comparison performance (Table in Section 4.4) is extended in a variable-controlling approach (Section 4.4). We also carried a test to compare our full-text level mechanism quantitatively (Table in Section 4.7).

The reminder of our work is organized as follows. We present related work in Section 2. Section 3 describes our models in detail. Subsequently, Section 4 shows experimental analysis, followed by the conclusion and future work in Section 5.

## 2. Related Work

In recent years, deep learning has shown great improvement in pattern recognition and other application fields [27,28,29,30]. In this section, we introduce the related techniques on the application of deep learning in the field of RS.

### 2.1. Attention Mechanism in RS

The idea of attention mechanism is inspired from the human’s ability to allocate attention on the observed objects according to tasks while ignoring the impact of others [31]. Thus far, the attention mechanism has been applied widely in many tasks of deep learning, such as information retrieval [32], recommendation [33] and machine translation [34]. The core of soft attention is to train an efficient and effective neural network to assign attentive weights for features vectors in an end-to-end manner. In the field of RS, He et al. [35] introduced an attention mechanism in CF to learn to assign both component-level and item-level attention weights on a set of features for multimedia recommendation. As for review text, Chen et al. [21] adopted attention mechanism to learn the usefulness of each review and extract the feature vector of users and items for predicting item ratings and generating explanation. In addition, Seo et al. [23] used convolutional neural networks with dual local and global attention networks to model user preferences and item properties. The attention layer selects keywords that account for to the rating by a local or global window. In this paper, we propose a multi-level attention mechanism that consists of word level attention layer and full-text attention layer to better model users and items for predicting item ratings.

### 2.2. Capturing High- and Low-Order User–Item Interactions

The coupling of FM and DNN has first introduced in click-through rate (CTR) prediction. DNN has shown effectiveness in CTR for learning sophisticated feature interactions [36]. Traditional approaches such as FM give a close approximation to the low-order feature interactions [11]. Many studies on the modeling of both high-order and low-order interactions [14,25,26] have been carried out. Cheng et al. proposed a network consisted of linear part and deep part to capture high- and low-order interactions. To leverage FM, Zhang et al. proposed FNN [25], which is a feed-forward neural network to model user–item interactions with an FM-initialized input. Based on the model mentioned above, Guo et al. proposed an end-to-end DeepFM model without any pre-train or feature engineering. DeepFM makes the “FM part” and “deep part” share the same input and shows impressive performance in CTR. In this paper, we follow the model of DeepFM and design a hybrid prediction structure for rating predictions. The main difference between our hybrid prediction structure and DeepFM is that the input of our model is the extracted feature vector from attention-based neural networks while that of DeepFM is the raw and sparse feature vector. Thus, our prediction structure does not include dense embedding layer, which enhances the memory efficiency of our model.

## 3. Our Approach

### 3.1. Overview

**Figure 1:**
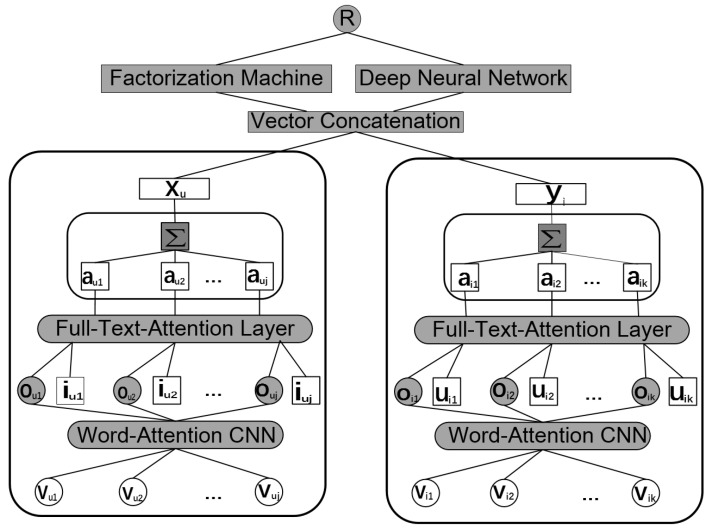
The architecture of MAHR.

To select both keywords and useful reviews, we utilize the multi-level attention mechanism to automatically assign weights to reviews and words when modeling users and items. Figure 1 illustrates the architecture of *MAHR*. The model contains two similar attention-based neural networks for users ($N_{et_{u}}$) and items ($N_{et_{i}}$) respectively, after which is the vector concatenation layer and the hybrid prediction layer. Note that the hybrid prediction layer consists of Factorization Machine and Deep Neural Network to capture the feature of concatenated vector more efficiently. Reviews written by the same users or on the same items are fed into to $N_{et_{u}}$ and $N_{et_{i}}$. The corresponding rating is calculated as the sum of FM and DNN (we can also use other methods to combine the output of FM and DNN). In the following subsections, due to the similarity between $N_{et_{u}}$ and $N_{et_{i}}$, we only give the details $N_{et_{i}}$. The same analysis is also practicable for $N_{et_{u}}$.

In the first stage of $N_{et_{i}}$ , the word-attention CNN Processor is applied to process the textual reviews of item *i*. In this processor, the network processes each review of item *i*, respectively. Specifically, each review is first transformed into a matrix of word vectors by the word-embedding technique, which we denote as $V_{i1}$, $V_{i2}$, ⋯, $V_{ik}$. Then, these matrices are sent to word attention CNN and the feature vectors of them can be obtained from the output. These feature vectors are denoted as $O_{i1}$, $O_{i2}$, ⋯, $O_{ik}$. Note that the keywords obtain higher weights in the word-attention CNN Processor, which will result more in precise feature vector of reviews than the normal CNN without word level attention mechanism.

The second stage of $N_{et_{i}}$ is the full-text-attention layer. The inputs are the feature vectors from word-attention CNN and the respective ID embedding of users who writes reviews for item *i*. In the full-text-attention layer, we calculate the contribution of each review and aggregate these vectors to get the representation of item *i*:

(1) $$\begin{array}{c} O_{i}=\sum_{l=1}^{k}a_{il}O_{il} \end{array}$$

where $a_{il}$ is the corresponding weight of the *l*-th review for item *i*, which will learn by the full-text-attention layer.

After we obtain the feature vector of item *i*, we pose a fully connected layer to customize the dimension of vectors according to the number of latent factors. The output of $N_{et_{i}}$ is the latent vector of item *i*, which is denoted as $Y_{i}$. Recall that the output of left network $N_{et_{u}}$ is the latent vector of user *u*, which is denoted as $X_{u}$.

The final structure is the prediction layer. In MAHR, we adopt the assumption of latent factor model that the rating of users to items are the results of the interaction of users’ and items’ latent vector. We extend the origin handle of user–item interaction by replacing the inner product with the coupling of FM and DNN. The objective function is the $\ell_{2}$-norm of recorded rating matrix. To avoid overfitting, we adopt the dropout technique to improve the generalization ability of MAHR.

### 3.2. Word-Level Attention-Based Mechanism

Inspired by the human visual attention, we further develop a word attention mechanism for reviews. When we read text or see images, we probably focus on a certain part of the input to understand or recognize them more efficiently. Generally, people tend to be attracted by the significant word in a local part. Here, we introduce a word-level attention module to measure the significance of one word.

Suppose *X* is a review with *T* word embeddings $\left(x_{1},x_{2},\cdots ,x_{T}\right)$ and $X_{att,i}$ is a slip window. Here, $x_{i}$ is the center word and *w* is the width of local window. The significance for each center word in window is given by:

(2) $$\begin{array}{cc} X_{att,i} & ={\left(x_{i+\frac{-w+1}{2}},x_{i+\frac{-w+3}{2}},\cdots ,x_{i},\cdots ,x_{i+\frac{w-1}{2}}\right)}^{T}\\ s_{i} & =g\left(X_{att,i}*W_{att}^{1}+b_{att}^{1}\right),\;\;\;i\in \left[1,T\right] \end{array}$$

where $s_{i}$ is the informative score of the *i*-th word and $W_{att}^{1}\in R^{w\times d}$ is a parameter matrix and $b_{att}^{1}$ is a bias. The * is the operation of element-wise multiplication and sum. The score can be direct used as a weight for *i*-th word embedding or we can apply a threshold to remove “trivial” words and only consider informative attention words. In this work, we use scores as weights. We use sigmoid for the activation function *g*. The weighted word embedding is:

(3) $$\begin{array}{c} {\tilde{x}}_{t}=s_{t}x_{t} \end{array}$$

where t∈[1,T]. To learn a global semantic representation, the weighted word embedding matrix $\tilde{X}$ is then fed into textual CNN module, which consists of a 1-D convolutional layer and max pooling layer. The output is the feature vector $O_{il}$ of the *l*-th review in item *i*.

The textual CNN module includes a convolutional layer and a max-pooling layer. Suppose the number of filters is $n_{att}$. Let $W_{j}^{2}\in R^{t\times d}$ be a filter and $b_{j}^{2}$ be a bias where $j\in [1,n_{att}]$. Then, $O_{il}$ is obtained by:

(4) $$\begin{array}{cc} z_{j} & =\mathrm{ReLU}\left(\tilde{X}⊙W_{j}^{2}+b_{j}^{2}\right),\;\;j\in \left[1,n_{att}\right]\\ O_{il} & =[\mathrm{Max}\left(z_{1}\right),\mathrm{Max}\left(z_{2}\right),\cdots \mathrm{Max}\left(z_{n_{att}}\right)] \end{array}$$

where ⊙ is the convolution operation and Max is the max-pooling operation. ReLU is a nonlinear activation function. The $\mathrm{Max}(z_{j})$ is the feature obtained by convolution filter $W_{j}^{2}$ and max-pool layer.

### 3.3. Full-Text Level Attention-Based Mechanism

The goal of the Full-Text Level Attention-based Layer in $N_{et_{i}}$ is to calculate the significance of one review for the features of item *i* and then aggregate all weighted reviews to characterize item *i*. A two-layer network is used for the attention score $a_{il}$. The input contains the feature vector of the *l*-th review of item *i* ($O_{il}$) and the user who wrote it (ID embedding, $u_{il}$). The ID embedding is added to model the quality of users, which helps identify users who always write less-useful reviews. Formally, the attention network is defined as:

(5) $$\begin{array}{c} a_{il}^{*}=h^{T}ReLU\left(W_{O}O_{il}+W_{u}u_{il}+b_{1}\right)+b_{2} \end{array}$$

where $W_{O}\in R^{t\times k_{1}}$, $W_{u}\in R^{t\times k_{2}}$, $b_{1}\in R^{t}$, $h\in R^{t}$, and $b_{2}\in R^{1}$ are parameters. The feature vector $O_{il}$ is obtained by the word-attention CNN. In addition, $u_{il}$ is calculated from one-hot code of user ID by the lookup operation in tensorflow. *t* denotes the size of hidden layer.

The final weights of reviews are predicted by the softmax function to normalize the above attention scores. The contribution of the *l*-th review to the final feature vector of item *i* is given by:

(6) $$a_{il}=\frac{exp(a_{il}^{*})}{\sum_{l=0}^{k}exp\left(a_{il}^{*}\right)}$$

After we obtain the attention weight of each review, the feature vector of item *i* is calculated as Equation (1).

Then, $O_{i}$ is sent to a fully connected layer with weight matrix $W_{0}\in R^{n\times k_{1}}$ and bias $b_{0}\in R^{n}$ to customize the dimension of latent vector. The final representation of item *i* is given by:

(7) $$\begin{array}{c} Y_{i}=W_{0}O_{i}+b_{0} \end{array}$$

### 3.4. Hybrid Prediction Layer

We aim to capture both linear and non-linear interactions between users and items. We develop a hybrid prediction layer in MAHR. As shown in Figure 1 , the prediction layer consists of two components, *FM component* and *Deep component*, which share the same input. First, let us concatenate $X_{u}$ and $Y_{i}$ into a single feature vector $z_{i}=\left(X_{u},Y_{i}\right)$. For the feature vector $z_{i}$, the predicted rating $\tilde{R}$ is given by:

(8) $$\tilde{R}=sigmoid\left(R_{FM}+R_{NN}\right)$$

where $R_{FM}$ and $R_{NN}$ are, respectively, the output of FM component and deep component.

The FM component is a factorization machine [11]. In addition to modeling first-order linear interactions among features, FM also uses a dot product between vectors to model a second-order pairwise feature interactions. The output of FM is the sum of a bias and the two kinds of interactions. $R_{FM}$ is given by:

(9) $$\begin{array}{c} R_{FM}=w_{0}+\sum_{i=1}^{\left|z_{i}\right|}w_{i}z_{i}+\sum_{i=1}^{\left|z_{i}\right|}\sum_{j=i+1}^{\left|z_{i}\right|}<v_{i},v_{j}>z_{i}z_{j} \end{array}$$

where $w_{0}$ is the global bias, $w_{i}$ measures the impact of the *i*-th variable in $z_{i}$ and $<v_{i},v_{j}>$ models the second-order interactions.

The deep component is a deep neural network for learning the high-order feature interactions. The architecture of the network is given by:

(10) $$\begin{array}{c} a^{\left(l+1\right)}=\sigma \left(W^{\left(l\right)}a^{\left(l\right)}+b^{\left(l\right)}\right) \end{array}$$

where *l* is the depth of neural network and σ is an activation function. $a^{\left(l\right)}$, $W^{\left(l\right)}$, and $b^{\left(l\right)}$ are the input, weight matrix, and bias of the *l*-th layer, respectively.

The prediction layer for $R_{NN}$ in deep component is given by:

(11) $$\begin{array}{c} R_{NN}=\sigma \left(W^{H+1}\right)a^{H}+b^{H+1} \end{array}$$

where *H* is the number of hidden layers and σ is the sigmoid function.

Based on DeepFM, all parameters, including $w_{i}$, $v_{i}$, and the network parameters ($W^{\left(l\right)}$ and $b^{\left(l\right)}$), are trained jointly for the combined prediction model.

### 3.5. Learning

The task that we focus on in this paper is rating prediction, which actually is a regression problem. For regression, a popular objective function is the squared loss. The objective function is given by:

(12) $$\begin{array}{c} J=\sum_{u,i\in \Omega }{\left({\tilde{R}}_{u,i}-R_{u,i}\right)}^{2} \end{array}$$

where Ω denotes the set of instances for training and $R_{u,i}$ is the rating assigned by user *u* to item *i*. To optimize the objective function, we adopt the Adaptive Moment Estimation (ADAM) as the optimizer. It can adjust the learning rate during the training phase, which avoids the process of choosing an efficient learning rate and results in the faster convergence than the SGD.

To alleviate overfitting, we consider dropout [37], a widely used method in deep learning models. Dropout stop working during testing and we use the whole network for prediction. Through dropout, we can prevent complex coadaptations of neurons on training data. Moreover, dropout may potentially improve the performance of the whole neural network due to the side effect of performing model averaging with smaller neural networks.

## 4. Experiment

We performed extensive experiments on three popular rating datasets with reviews to verify the effectiveness of MAHR model in comparison with other state-of-the-art approaches. We first give the details of experimental settings in Section 4.1, including datasets description, comparison methods, evaluation metric, and parameter settings. The experimental methodology is presented in Section 4.2, followed by a parameter sensitivity analysis in Section 4.3. Besides, we present the performance comparison in Section 4.4. In Section 4.5 and Section 4.6, we present the validation of the effectiveness of multi-level attention mechanism and hybrid prediction structure, respectively. Afterwards, the explainability analysis is presented.

### 4.1. Experimental Settings

#### 4.1.1. Datasets

We tested MAHR methods on three popular datasets. The first dataset is *Yelp* Challenge 2015 (https://www.yelp.com/dataset/challenge), a dataset for restaurant ratings and comments. Yelp contains more than one million reviews and thirty thousand users. The second and third dataset are both from *Amazon* (http://jmcauley.ucsd.edu/data/amazon/) product data with five cores [38]: *Books* (8,898,041 reviews and 22,507,155 ratings) and *Electronics* (1,689,188 reviews and 7,824,482 ratings). These two datasets were selected to cover the most classic scenarios for online services and applications, i.e., reading books and online shopping.

Table 1 shows the statistics of three datasets. In Table 1, we can find that the three datasets have different sparsity in rating matrix. All of these datasets have more than one million reviews, while users in Yelp and Electronics provide fewer reviews than Books on average, which indicates that Yelp and Electronics are much sparser than Books. As mentioned in the Introduction, the sparsity problem could deteriorate the prediction performance of recommender systems. Note that the lengths of reviews in all datasets are less than 150 words on average.

#### 4.1.2. Comparison Methods

To show the superiority of our MAHR method, we selected seven comparison methods: Probabilistic Matrix Factorization (PMF) [5], Non-negative Matrix Factorization (NMF) [39], Latent Dirichlet Allocation (LDA) [40], Collaborative Topic Regression (CTR) [41], Deep Cooperative Neural Networks (DeepCoNN) [19], Neural Attentional Regression model with Review-level Explanations (NARRE) [21], and dual attention-based model (D-Attn) [23]. These methods can be divided into three categories: (1) traditional CF model without leveraging review (PMF and NMF); (2) topic modeling based approaches with the application of review information (LDA and CTR); and (3) deep recommender systems with the utilization of review (DeepCoNN, NARRE, and D-Attn). The first category was the blank group to validate whether the review information is helpful to for recommender system. The second category was the topic-model group to compare deep recommender model with topic modeling based recommender systems. The third category was the deep-model group to compare our MAHR model with other deep RS with review information. The topic-model group and deep-model group import the review information to improve the prediction performance. The characteristics of the comparison methods are listed in Table 2.

- PMF: Probabilistic Matrix Factorization models the rating matrix as the product of two lower-rank user and item matrix with a Gaussian error distribution and obtains good performance on the large, sparse, and very imbalanced datasets.
- NMF: Non-negative Matrix Factorization is another useful matrix factorization with only rating matrix involved.
- LDA: Latent Dirichlet Allocation is a famous topic modeling algorithm. By employing LDA on review text, we can learn a topic distribution for each item and obtain the topic preference for each user.
- CTR: Collaborative Topic Regression combines CF with topic modeling in a probabilistic model, which show better performance than matrix factorization methods.
- DeepCoNN: Deep Cooperative Neural Networks learns hidden latent features for users and items jointly using two coupled neural networks. The prediction layer introduces Factorization Machine as the estimator of the corresponding rating. DeepCoNN is a state-of-the-art method in deep RS that utilize review information.
- NARRE: Neural Attentional Regression model with Review-level Explanations use a novel attention mechanism to assign weights to reviews, which learn latent features for users and items jointly using two parallel neural networks. The prediction layer is based on the Latent Factor Model. NARRE is a novel deep RS model with full-text level attention mechanism.
- D-Attn: Dual Attention-based model is an advanced deep RS model with dual word level attention mechanism. The weights of words are learned from a local or global window. The local window extracts keywords and the global one selects noisy words. The predicted rating is calculated by the inner product of user feature vector and item vector.

#### 4.1.3. Parameter Settings

The dataset was randomly split into training set (80%), validation set to tune hyper-parameters (10%), and test set (10%). The hyper-parameter for comparison methods were initialized according to the corresponding papers and tuned based on the performance on the validation set to obtain optimal performance.

For PMF and NMF, we selected the optimal parameter for the number of latent factors from {10,20,40,60,80,100}, and regularization parameter from {0.001,0.01,0.1,1.0}. For LDA and CTR, we chose the number of topics from {5,10,20,50,100}. After searching, the number of latent factors for PMF and NMF were set to 60 and the number of topics for LDA and CTR was 10. Following the experiments in [19], the hyper-parameters for CTR were: α=0.1, $\lambda_{u}=0.02$ and $\lambda_{v}=10$.

For deep models, we reused the parameters for deep neural network reported in the corresponding papers. Moreover, Google News was imported as a pre-trained word embeddings and the dimension of word embedding is 300. For MAHR model, we searched the dropout ratio from {0.1,0.3,0.5,0.7,0.9} and the number of hidden layer from {1,2,3,4}. The number of latent factors was searched from {10,20,40,60,80,100}. The number of hidden layer was 2 and the dropout ratio was 0.5. The window size for the word attention layer was 5 and the number of convolutional kernels was 100. The latent factor number was 60.

#### 4.1.4. Evaluation Metric

In the experiments, we adopted the Root Mean Square Error (RMSE) to evaluate the prediction performance of our MAHR model. RMSE has been used in plenty of works on RS, including for both traditional and deep learning methods [5,21]. RMSE can be defined as follows:

(13) $$\begin{array}{c} RMSE=\sqrt{\frac{1}{\left|\mathrm{TEST}\right|}\sum_{u,i\in \mathrm{TEST}}{\left(R\left(u,i\right)-\hat{R}\left(u,i\right)\right)}^{2}} \end{array}$$

where TEST is the set of observation.

### 4.2. Methodology

We followed a four-step methodology to demonstrate the performance of our proposed MAHR model:

- To explore the optimal parameter of MAHR model and the parameter sensitivity, we carried out a parameter study on the validation set. We evaluatd our approach based on the optimal parameters.
- To analyze the prediction performance of MAHR, we compared the RMSE of different methods based on the test set. We made some observations based on the results.
- To validate the effectiveness of MAHR model, we produced some variant MAHR models (MAHR-multi, MAHR-word, MAHR-full-text, MAHR-non, MAHR-hybrid, MAHR-fm, MAHR-nn, and MAHR-dot-product) by controlling the application of multi-level attention mechanism and hybrid prediction structure. We also observed the advantage of our methods in comparison with variant methods.
- To show the explainability, we visualized our multi-level attention layers and the corresponding weights. We also calculated the prediction and recall of our full-text level attention for predicting useful reviews on Amazon datasets (Books and Electronics). The comparison results with simple strategies (random, latest, and longest) are reported.

### 4.3. Parameter Sensitivity Analysis

Based on the Yelp and Books dataset, we analyzed the influence of different hyper-parameters of traditional approaches and deep models including DeepCoNN, NARRE, D-Attn, and MAHR. The order was: (1) dropout rate; (2) number of hidden layers; and (3) number of latent factors.

**Figure 2:**
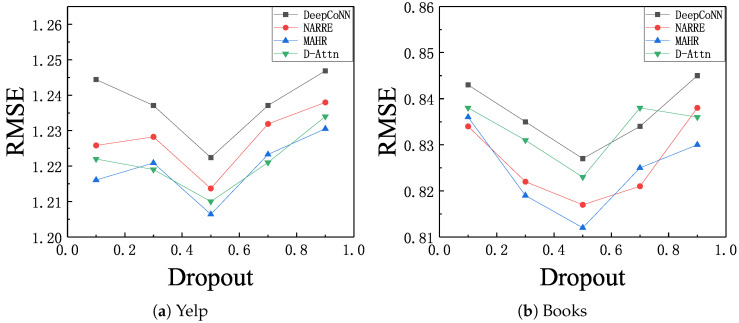
RMSE with respect to different dropout ratios, (**a**) Yelp; (**b**) Books.

We first studied the impact of the drop rate. We searched the dropout from {0.1,0.3,0.5,0.9}. The results of deep models with respect to different dropout ratios are shown in Figure 2. we found that all methods benefited from a proper value of dropout ratio and could reach the peak of prediction performance when dropout rate was 0.5, which demonstrates that the dropout technique could prevent overfitting and improve the prediction accuracy. Basically, all of these curves witnesses a decrease in RMSE as the dropout rate oncreased after 0.5. These results prove that a proper dropout rate could enhance the robustness of model.

In addition, we also observed that the RMSE curve on Yelp dataset changed more sharply than that on Books dataset, which indicates the prediction accuracy of Yelp dataset was more sensitive to dropout rate than the prediction accuracy of Books dataset. We think the difference in the parameter sensibility was caused by the difference on the sparsity of dataset, given the common phenomenon in deep learning that a small dataset tends to be more likely overfit without dropout.

**Figure 3:**
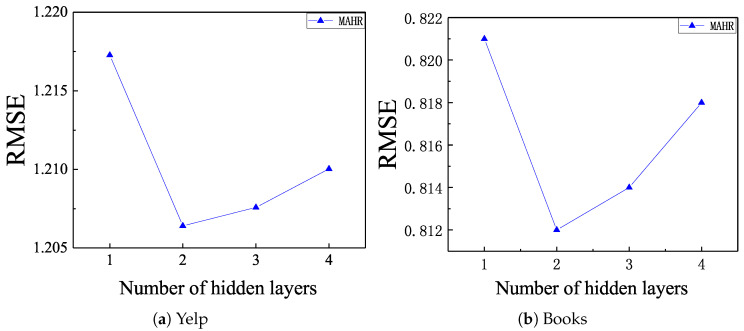
RMSE with respect to different hidden layers, (**a**) Yelp; (**b**) Books.

We then studied the effect of the number of hidden layers in hybrid prediction structure. Because the DeepCoNN, NARRE, and D-Attn have no hidden layers in prediction layer, we only carried out the second parameter study on MAHR. As presented in Figure 3, increasing number of hidden layers in the hybrid prediction layer raised the accuracy of the models at the beginning. However, when we kept increasing the number of hidden layers, their performance degraded, as a result of overfitting.

**Figure 4:**
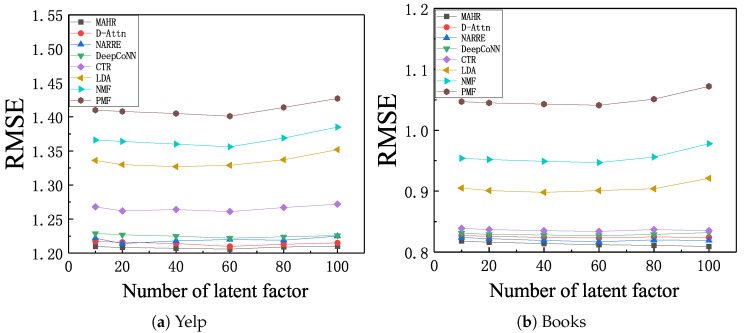
RMSE with respect to different latent factors, (**a**) Yelp; (**b**) Books.

We finally explored the effect of the number of latent factors. The results are shown in Figure 4 For deep models group (DeepCoNN, NARRE, D-Attn, and MAHR), the number of latent factors was equal to the dimension of input vector for prediction layer. Generally, we observed that the RMSE curves of all deep methods showed a gentle and trivial change while the blank group (PMF and NMF) and topic model group (LDA and CTR) had a bigger change than deep model group. This demonstrates the benefits of deep learning for extracting the feature of review text. We found that MAHR achieved the best RMSE performance on both datasets and under all latent factor numbers, in comparison with other deep models, which shows the advantages of multi-level attention mechanism and hybrid prediction layers.

### 4.4. Performance Comparison

The performance of MAHR and the baselines are reported in terms of RMSE in Table 3. The best performance is shown in bold and the averages on three datasets are reported. From the results, several observations can be made.

First, we found that both traditional matrix factorization methods (PMF and NMF) in blank group did not obtain comparable performance to those methods in the control group that utilize reviews. The gap in performance between blank group methods and control group methods validated our hypothesis that review text could supply additional information and considering reviews in models further improve the accuracy of rating prediction.

Secondly, although the simple employment of topic modeling methods (LDA and CTR) to learn topic characters from item reviews could improve the performance of recommendation system, deep models group better captured the feature of review when compared with topic modeling group. By modeling ratings and reviews together and using supervised learning for regression tasks, DeepCoNN, NARRE, and MAHR obtained additional improvements. In these experiments, LDA modeled reviews without the feedback from users ratings. Thus, the learned unsupervised features from LDA might be not as efficient as the expectation. Compared with LDA, CTR obtained additional improvements by jointly modeling reviews and rating. However, CTR could not beat deep methods on all datasets because of the weaker capacity of jointly modeling and extracting semantic features.

Thirdly, as shown in Table 3, our method MAHR consistently outperformed all baseline methods. Although review information was useful in recommendation, the performance varied depending on how the review information was utilized. Our model proposes a multi-level attention mechanics for extracting both word-level and full-text-level information. This allows a review to be modeled with a finer granularity, which can lead to a better performance according to the results. Compared to PMF, our approach gained 30.06% improvement on average. In Table 3, all methods obtained better performance on Book dataset than on Yelp and Electronics. We think it was caused by the sparsity of Yelp and Electronics. We found that the method using review information could effectively alleviate the sparsity problem on all datasets, which validates the hypothesis that review information can help model user and item, leading to additional improvement.

### 4.5. Effect of Multi-Level Attention

**Figure 5:**
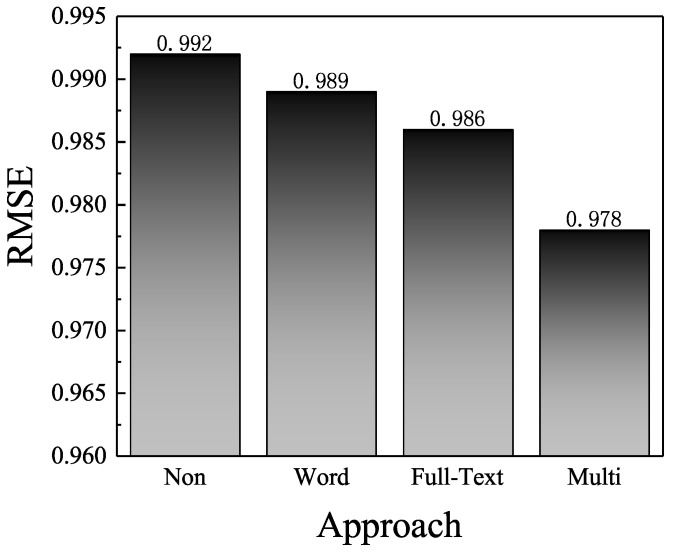
Effect of attention mechanism.

We next focused on validating the effeteness of multi-level attention mechanism. We produced several variant MAHR models through assigning normalized constant weights. The variant MAHR models include the original MAHR model with multi-level attention mechanism (MAHR-multi), the MAHR model with word level attention mechanism (MAHR-word), the full-text level attention mechanism one (MAHR-full-text), and the model without any attention mechanism (MAHR-non). Note that, when we did not use full-text-level attention mechanics, a normalized constant weight was assigned to each review, and, when we did not consider the word-level attention, the Word-Attention degenerated to a normal textual CNN module. For MAHR-word (MAHR-full-text) model, we assigned normalized constant weights to reviews (words). For MAHR-non, we assigned constant weights to both words and reviews. In Figure 5, we compare the average RMSE on three datasets.

In the figure, we make several observations. First, all three methods with attention layer (MAHR, MAHR-word, and MAHR-full-text) outperformed MAHR-non model, which did not apply any attention mechanism. No matter what type the attention mechanism, the prediction accuracy benefited from the extracted information in review text, which justifies the common assumption that the significance of different reviews and words vary and proper modeling of different weights results in additional improvement. Second, the multi-level attention approach made the most precise prediction, which validates the usefulness of our approach. From the better performance of the full-text level approaches compared to the word-level one, we found that the full-text-level attention has a more significant contribution to the improvement. Third, the average RMSE of MAHR-non model was lower than that of DeepCoNN, which indicates the prediction accuracy of MAHR without any attention mechanism is better than the accuracy of DeepCoNN. This improvement was probably caused by the effective hybrid prediction layer in MAHR.

### 4.6. Effect of Hybrid Prediction

**Figure 6:**
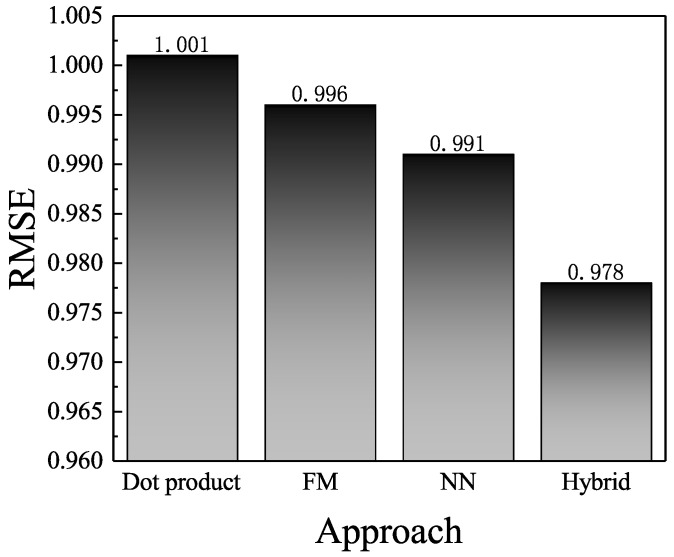
Effect of hybrid prediction layer.

In this section, our controlled experiment on the effect of hybrid prediction layer is presented. Three new variants were used. The first one (MAHR-dot-product) is the traditional dot-product prediction layer: $\tilde{R}=X_{u}Y_{i}$. The last two approaches use FM (MAHR-fm) or NN(MAHR-nn) solely as prediction layer. We implemented the MAHR-dot-product model by switching off the hybrid prediction layer and replacing the vector concatenation layer with the dot-product output layer. As for MAHR-fm and MAHR-nn, we implemented them by switching off the Deep Neural Networks and Factorization Machine, respectively. We reused the hyper-parameter setting of MAHR model in the last two variants. The average RMSE on three datasets are shown in Figure 6.

As shown in Figure 6, the MAHR-dot-product obtained the worst performance in all methods. The difference in RMSE justifies that the simple dot product for prediction layer might miss information about the non-linear interaction between users and items and lead to a loss in prediction accuracy. Furthermore, we found that the MAHR-hybrid made the most precise prediction, which validates the usefulness of our hybrid prediction layer. When compared with MAHR-FM, MAHR-NN obtained higher prediction accuracy. We think the reasons were as follows. First, the deep neural networks could capture the non-linear user–item interactions, while our implemented FM could only model the second-order interactions, which is the major limitation of prediction layer. Second, the dropout technique in deep learning could avoid potentially overfitting and obtain additional improvement. In addition, the RMSE of MAHR-dot-product was apparently lower than that of LDA and CTR, which also use the inner product of latent vectors to predict rating. The decrements in RMSE probably benefited from the robust multi-level attention mechanism.

### 4.7. Explainability Analysis

#### 4.7.1. Word Level Explanation

**Figure 7:**
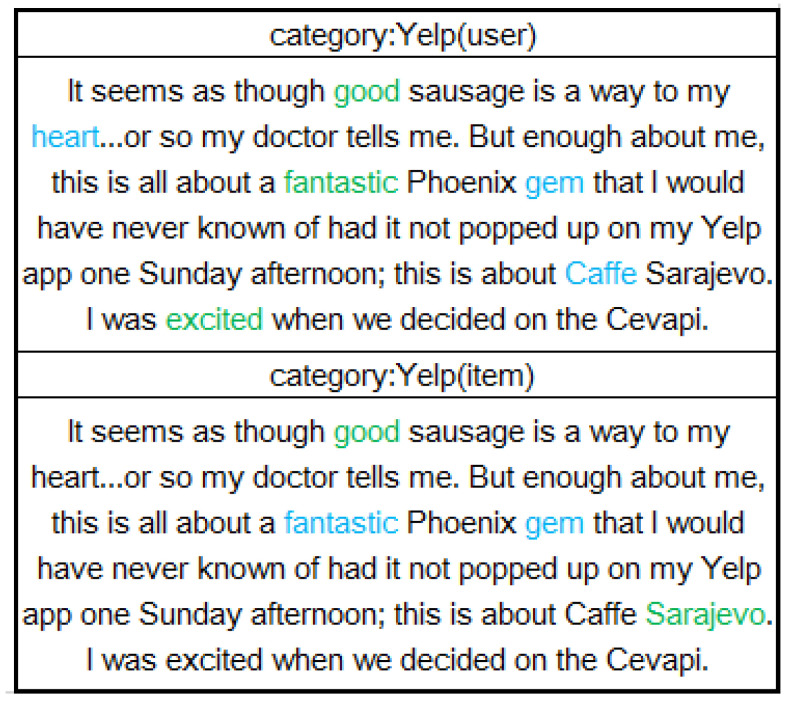
Word level explanation analysis through visualization of keywords with high weights.

To validate our design on the word-level attention module, we highlighted keywords with high weights in the attention module. Colored words were considered as informative words, and green words had higher attention scores than those of blue words. We randomly selected the same review from Yelp but highlighted differently by the user network and the item network in Figure 7.

We made two key observation. First, similar keywords were highlighted in the user network and item network. All of these keywords were likely words that describe properties of the item or some more personalized words. However, the two networks chose different attentional words, because the two networks were trained with different sets of reviews and the network decided the keywords by reviews and ID embedding. For example, the user network gave high scores to keywords expressing subject feelings, e.g., “good”, “excited” and so on. The item network tended to focus on nouns that describe properties of an item.

#### 4.7.2. Full-Text Level Explanation

**Figure 8:**
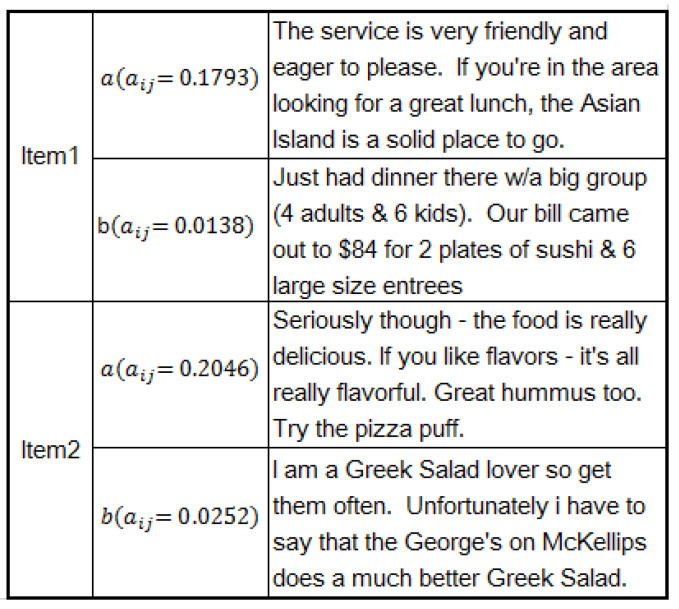
Full-text level explanation analysis through visualization of reviews with high weights.

To analyze the explainability of full-text-level attention module, we first provide some reviews and their final attention weights in Figure 8 and then compare the prediction performance of full-text-level attention module in Table 4.

Figure 8 shows examples of the high-weight and low-weight reviews selected by our model, where $a_{ij}$ means the weight of attention. The first reviews for Item 1 and Item 2 had higher weights, while the second reviews for Item 1 and Item 2 were less helpful reviews with lower weights. Generally, the reviews with high attention weight contain more information about the item. For example, the buyers can easily get the feature of each item from Reviews 1a and 2a, which is highly instructive for making purchasing decisions. In contrast, the low attention reviews only contain the authors’ general opinions, but give fewer details to help make a decision.

We carried out a prediction and recall test on the Books and Electronics datasets, which contain some reviews that have been rated useful by other users. We assumed that the rated reviews are ground truth to study the performance of full-text-level attention module. We only reserved the items having at least one rated review. We selected three comparison methods: Latest (the rated reviews list are generated by selecting the latest *K* reviews), Random (the rated reviews list are generated randomly), and Length (the rated reviews list are generated by selecting the longest *K* reviews). Our MAHR selected *K* reviews with the highest weights. We calculated the precision and recall for a review list with *K* reviews according to *Precision*@*K* and *Recall*@*K* in [21]. *Precision*@*K* and *Recall*@*K* are as follows:

(14) $$\begin{array}{c} Precision@K=\frac{\sum_{j=1}^{K}rel_{j}}{K};\;\;Recall@K=\frac{\sum_{j=1}^{K}rel_{j}}{Re_{i}^{rated}}; \end{array}$$

where $rel_{j}$ = 1/0 indicates whether the No.j review in the Top-*K* list is rated helpful. The $Re_{i}^{rated}$ is the number of rated reviews in item *i*. To evaluate the effect of length of review list, we set *K* = 1 and 10.

In Table 4, we can find that the precision and recall of MAHR were impressively better than that of the three other methods. It shows that the weights obtained by full-text-level attention module are consistent with the users’ needs and perceptions. By applying the full-text level attention mechanism, the significance of different reviews can be learned effectively.

## 5. Conclusions and Future Work

In this paper, We propose a Multi-level Attentional and Hybrid-prediction-based Recommender (MAHR) model that not only leverages the hybrid prediction structures to replace simple inner product of two latent vectors, but also innovatively implement a multi-level attention mechanism to combine word level significance and full-text level usefulness. It selects both useful words and reviews automatically to provide word-level and full-text-level explanations and make a more precise prediction. In addition, it models the non-linear interaction between user and item in a hybrid prediction layer, which couples the factorization machine to a deep neural network. Extensive experiments were made on three real-life datasets from Amazon and Yelp. The visualization and analysis of keyword and useful reviews validated the reasonability of our multi-level attention mechanism. In terms of recommendation performance, the proposed MAHR consistently outperformed the state-of-the-art recommendation models based on matrix factorization and deep learning in rating prediction. We believe this work offers a new approach to capture the context of recommendation systems.

In the future, we plan to combine the transfer learning and the latent factor model to build a more robust prediction layer for recommender system. Moreover, we are interested in the exploration of more advanced neural networks, e.g., Long Short-Term Memory (LSTM) network, which use sequence learning, to handle sequence and sentiment analysis in the review texts. 

## Figures and Tables

**Table 1:** Statistics of datasets.

Dataset	Yelp	Books	Electronics
# of Users	366,715	708,955	786,330
# of Items	60,785	33,122	61,896
# of Reviews	1,569,264	8,898,041	1,689,188
Avg. # of Words per a review	126.41	113.23	120.67
Avg. # of Reviews per a user	4.3	12.55	2.15
Avg. # of Reviews per an item	25.8	268.6	27.3

**Table 2:** Characteristics of the comparison methods.

Characteristics	PMF	NMF	LDA	CTR	DeepCoNN	NARRE	D-Attn	MAHR
Ratings	✔	✔	✔	✔	✔	✔	✔	✔
Textual Reviews	×	×	✔	✔	✔	✔	✔	✔
Deep Learning	×	×	×	×	✔	✔	✔	✔
word level attention	×	×	×	×	×	×	✔	✔
full-text level attention	×	×	×	×	×	✔	×	✔

**Table 3:** RMSE comparison for different methods.

	Yelp	Books	Electronics	Average on all Datasets
**PMF**	1.401	1.041	1.373	1.272
**NMF**	1.356	0.947	1.092	1.132
**LDA**	1.327	0.898	1.012	1.078
**CTR**	1.261	0.835	0.972	1.022
**DeepCoNN**	1.222	0.827	0.933	0.994
**NARRE**	1.214	0.817	0.921	0.984
**D-Attn**	1.210	0.823	0.928	0.987
**MAHR**	**1.206**	**0.812**	**0.915**	**0.978**

**Table 4:** Prediction and recall of full-text-level attention module on Books and Electronics.

	Books				Electronics			
	Latest	Random	Length	MAHR	Latest	Random	Length	MAHR
Precision@1	0.1561	0.3418	0.2600	0.4053	0.2569	0.4803	0.4243	0.5497
Recall@1	0.0380	0.1000	0.0810	0.1468	0.0420	0.1042	0.0895	0.1188
Precision@10	0.1628	0.2100	0.2432	0.2832	0.2339	0.2842	0.3080	0.3707
Recall@10	0.4585	0.6051	0.7101	0.9031	0.4736	0.5829	0.6476	0.8733
